# Predictors of outcome following a body image treatment based on acceptance and commitment therapy for patients with an eating disorder

**DOI:** 10.1186/s40337-022-00615-9

**Published:** 2022-07-01

**Authors:** Maria Fogelkvist, Sanna Aila Gustafsson, Lars Kjellin, Thomas Parling

**Affiliations:** 1 University Health Care Research Center, Faculty of Medicine and Health, Örebro University, 701 82 Örebro, Sweden; 2 Centre for Psychiatry Research, Department of Clinical Neuroscience, Karolinska Institutet, and Stockholm Health Care Services, Region Stockholm, The Centre for Psychotherapy, Education and Research, Liljeholmstorget 7, 117 63 Stockholm, Sweden

## Abstract

**Background:**

It is important to target body image in individuals with an eating disorder (ED). Acceptance and commitment therapy (ACT) has been trialed in a few studies for individuals with an ED. Although ACT outcomes in ED patients hold promise, studies of predictors are scarce. The aim of the present study was to explore differences in ED symptom outcome at two-year follow-up in subgroups of participants attending either treatment as usual (TAU), or a group intervention based on ACT targeting body image. Additionally, we aimed to compare subjective recovery experiences between groups.

**Methods:**

The study took place at a specialized ED outpatient clinic, and included patients diagnosed with an ED that had received prior treatment and achieved a somewhat regular eating pattern. Study participants were randomly assigned to continue TAU or to participate in a group intervention based on ACT for body image issues. Only participants that completed the assigned intervention and had completed follow up assessment by two-years were included. The total sample consisted of 77 women.

**Results:**

In general, ACT participants showed more favorable outcomes compared to TAU, and results were more pronounced in younger participants with shorter prior treatment duration and lower baseline depression ratings. Participants with restrictive ED psychopathology had three times higher ED symptom score change if participating in ACT in comparison to TAU.

**Conclusions:**

An ACT group intervention targeting body image after initial ED treatment may further enhance treatment effects. There is a need for further investigation of patient characteristics that might predict response to body image treatment, particularly regarding ED subtypes and depression ratings.

## Plain English Summary

Body image issues are common in patients with an eating disorder. Issues with body image are theorized to maintain eating disordered behaviors and are associated with an elevated risk of relapse after successful treatment. Targeting issues with body image in treatment programs for eating disorders might enhance treatment outcome and reduce risk of relapse. In this study, participants had either received treatment specifically targeting body image issues, or treatment as usual (TAU) at a specialized eating disorder center. The objective of the study was to explore if outcome regarding eating disorder symptoms differed by patient characteristics such as age and symptoms of depression, depending on treatment type. Results showed that patient characteristics were significant predictors for outcome. For example, in patients with binge eating and/or purging behaviors outcome did not differ depending on treatment, they improved from both. However, patients with a younger age, shorter duration of prior treatment, and lower depression ratings showed more pronounced benefits from the body image specific treatment than from the usual eating disorder treatment.

## Background

Preoccupation with body image has been suggested to be a core psychopathology in all eating disorders (ED; [11, 36]). This preoccupation is theorized to be the main factor that drives and maintains the ED, leading to behaviors that aim to control weight, shape and eating habits. Further, body image preoccupation that remains following ED treatment has been shown to increase risk of relapse [25]. There are no agreed upon criteria for defining recovery that consider the views from patients, clinicians, and researchers [32]. Since body image disturbance is part of the diagnostic criteria for both anorexia nervosa (AN) and bulimia nervosa (BN) [2], continued body image preoccupation might be viewed as incomplete recovery [4, 25]. However, as pointed out by Keel et al. [25] lower levels of preoccupation might also be viewed as “normative discontent” [37]. It is therefore important to address body image during ED treatment, and the addition of interventions targeting body image might aid patients reaching full recovery [31].

There are different approaches to body image treatment that depends on the theoretical approach to the suffering of the individual. Acceptance and commitment therapy (ACT) is a transdiagnostic approach to human suffering that posits that suffering is part of being human, and can be amplified as a consequence of efforts to alleviate it [17]. For patients with an ED and body image issues, the body may be viewed as a problem to be fixed. In their efforts to alleviate the suffering connected with body image issues, they endorse in ED behaviors such as restrictive eating, compulsive exercise, constant body checking, binge eating, and purging. These behaviors result in additional suffering as their ED worsens. There are only a few studies published on ACT for ED. These include small case studies for patients with AN [5, 18] and binge eating disorder [20]. Further, studies comparing ACT to treatment as usual (TAU) in specialized eating disorder centers, suggest that ACT may be as effective at reducing ED symptoms as TAU in outpatient care [12, 35], and adding ACT to TAU in residential care might enhance outcome [23].

When assessing treatment outcome in randomized controlled trials (RCT), it is important to consider if there might be baseline patient characteristics that influence treatment outcome. Such knowledge might provide clinicians with guidance as to which intervention might fit which patient. For patients with an ED, early symptom change predict favorable outcome [30, 41], while symptom severity predicts worse outcome and dropout [41]. However, since outcome definitions vary between studies the predictors will vary as well [8]. Few studies have investigated predictors of body image treatment or ACT treatment for ED. Legenbauer et al. [28] investigated predictors for body image treatment in patients with an ED. Changes in cognition related to body image and self-esteem, dietary restraint, eating and loss of control, internalization, and social comparison were examined. They found that reductions in these thoughts during treatment were associated with reductions in ED pathology. In a study comparing ACT to TAU for patients with an ED, more severe ratings of ED symptom at baseline predicted greater reductions in ED pathology for patients receiving ACT than TAU [22]. Furthermore, authors highlighted the need to understand which patients benefit from specific interventions, to make it possible to tailor interventions to individual patients.

A recently published study compared an ACT group intervention, targeting body image issues, to TAU, for patients with residual ED symptoms [12]. Results indicated that ACT was more effective at reducing ED psychopathology, according to self-assessment of ED symptoms and body dissatisfaction, as well as in enhancing mindful awareness, while requiring less specialized ED care at follow up. In the present study, we wanted to explore whether there were any patient subgroups or factors that could predict better or worse outcome. Research on outcome predictors for body image interventions in patients with ED is scarce. The aim of the present study was to explore differences in ED symptom outcomes at a two-year follow-up in subgroups of participants attending either TAU, or a group intervention based on ACT targeting body image. Additionally, we aimed to compare subjective recovery experiences between groups.

## Method

The present study builds upon data from an RCT (registered at Clinical Trials, ID: NCT02058121) that took place at a specialized ED outpatient treatment center in Sweden from 2010 until 2014. A power analysis for finding significant differences between the groups suggested the inclusion of 120 patients. No power analysis for finding predictors for treatment outcomes was made.

### Participants

Inclusion criteria were diagnosis of an ED according to DSM-IV, achievement of a somewhat regular eating pattern (defined as eating at least three meals per day, although still restrictive), and an age of 16–50 years. Exclusion criteria included severe psychiatric conditions that implicated need for more intensive treatment. Only women participated in this study although both men and women were invited.

### Procedure

Patient intake procedures at the current ED treatment center included ED pathology assessment via a structured ED interview (SEDI; [7]), and by self-assessment questionnaires. Treatment was offered individuals assessed as fulfilling diagnostic criteria for an ED according to DSM-5 [2]. At the beginning of recruitment, the DSM-5 had not yet been implemented nationwide in Sweden. Thus, participants were assessed according to DMS-IV criteria and their diagnoses were translated afterwards according to the DSM-5. Posters in the waiting room at the ED center advertised the treatment study. Twice a year all therapists at the treatment center reported eligible patients to the head investigator, who sent study information by mail. Responders were invited to an individual meeting where they received written and verbal information, were assessed and consented participation. When baseline assessments for the study had been collected, patients were randomized to either an ACT-based group intervention or TAU at the specialized ED treatment center. The randomization sequel was computer generated in blocks and provided by an independent statistician. The results of the randomization were given to participants in an enclosed envelope. Participants were reassessed at the end of treatment (approximately 16 weeks after baseline), and at follow-ups at one and two years after initial assessment. The present study included assessments at baseline (T1) and follow-up by 2 years (T4). The study was approved by the Uppsala Regional Ethical Review Board (Dnr. 2009/294, 2009-11-18).

### The ACT intervention

The ACT intervention in this study was a manualized application of a self-help book, *Lev med din kropp* [Live with your body] [14]. The book contains seven steps, and each step was covered in one or two treatment sessions. Treatment was given in a group setting, consisting of 12 weekly main sessions and one individual session before and after the group sessions. The current ACT treatment was tailored specifically for body image issues. Participants were prompted to do homework between sessions, which included mindfulness exercises, reading from the book, and completing other exercises in accordance with the book. Behavioral experiments were used to explore how their evaluative stance towards their bodies had an impact on their behavior; Do they avoid situations that provoke uncomfortable inner experiences regarding their bodies? Do they check their bodies in different ways? At treatment start participants made inventory of important areas in their lives and explored to what extent they were living a life they value. What hinders them from moving towards their values? Reactions to thoughts were explored; do they act on their thoughts as literal truths? In ACT, participants learn other ways of relating to their thoughts and to act in accordance with identified values. Rather than listening to evaluative thoughts that suggest continued avoidance of situations that provoke inner painful experiences, they were prompted to do the opposite if it is in line with what they value. A more detailed description of the intervention has been published previously [13].

### TAU

At the ED outpatient treatment center, TAU includes interventions that aim at normalizing eating patterns and restoring or stabilizing weight. Interventions are given individually or in group format and includes cognitive behavior therapy, interpersonal therapy, counseling, and seeing a physician, dietician or physiotherapist. The interventions are tailored individually based on diagnosis and decided upon in collaboration with the patient. All patients that were included in the present study had received TAU before randomization. The amount of TAU differed between patients both before and after randomization.

### Assessment

#### Self-report measures

***Eating disorder examination questionnaire*** (EDE-Q; [10]) The EDE-Q is a self-report questionnaire assessing ED symptoms during the past four weeks. It consists of 28 questions, where a global mean can be calculated based on 22 of the items, with responses ranging from 0 to 6. Higher values indicate more severe symptoms. The 22 items can be subdivided into four categories; assessment of dietary restraint in an effort to change or control weight or shape, the impact of eating concern on levels of anxiety and preoccupation with eating, and the level and impact of shape and weight concerns. The six remaining items investigate the frequency of common ED behaviors in the past four weeks, such as binge-eating, self-induced vomiting, laxative use, and compulsive exercise. The global score at T4 was the dependent variable in this study. A cut-off for a Swedish sample has been suggested at 2.76 [9]. Furthermore, a reliable change index (RCI; [42] of 1.45 on EDE-Q has been derived from a Swedish adult ED population [9]. An RCI can be used to calculate whether score changes are above expected measurement errors. Cronbach’s alpha for EDE-Q in the present study was 0.84.

***Montgomery-Åsberg Depression Rating Scale, the self-rating version*** (MADRS-S; [34, 39]) The MADRS-S is a self-report questionnaire assessing the level of depressive symptoms during the last three days. It consists of nine questions, with responses ranging from 0 to 6, where higher values indicate more severe depressive symptoms. A global score can be computed, ranging from 0 to 54. The global score can be used to suggest severity level of depression: no depression (0–12), mild depression (13–19), moderate depression (20–34) or severe depression (> 34) [38]. Cronbach’s alpha for MADRS-S in the present study was 0.88.

***Subjective experience of recovery*** At the end of follow up, all participants were asked to write down the answer to the following question regarding their recovery: “Do you perceive yourself as fully recovered from your eating disorder”? Answers were coded and dichotomized into YES or NO.

### Stratified analyses

Participants were divided into different subgroups depending on characteristics at baseline. Since the sample size was small and separated by treatment type, we decided that subgroups should not contain more than two categories. For each specified subgroup, categories were split by cut-offs that were deemed clinically meaningful. One subgroup was ED type, where participants were categorized into those who displayed a restrictive ED (AN spectrum) or displayed more binge eating and/or purging behaviors (BN spectrum), as suggested by e.g. Juarascio et al. [22]. Subgroups were created based on age (< 25/ ≥ 25), separating adolescents and young adults from older adults in accordance with the proposed age group of emerging adulthood [3]. For depressive symptoms MADRS-S was used, with a suggested cut-off score of moderate severity (≥ 20) [38], and ratings of ED symptoms according to cut-off (2.76) [9] on EDE-Q. Length of current treatment episode was divided by either longer or shorter than one year.

### Statistical analysis

All analyses were conducted using SPSS version 25. The outcome variable was EDE-Q score at T4, where 13% of data was missing. A prior study included change in outcome for all four assessments [12], and showed that differences between ACT and TAU were most pronounced at T4. Missing data were analyzed to ensure that it was missing completely at random (MCAR). This was done by both t-test investigations of possible differences in baseline characteristics and by pattern analysis of missing values. Data were stratified into subgroups by dichotomization described above. Dichotomization is common in prognostic studies and was decided upon since the manuscript is exploratory, and cutoffs were selected based on standardized scores [16]. Differences in score changes on EDE-Q from T1 to T4 between ACT and TAU were explored for the subgroups by running t-tests. Differences in subjective experience of recovery (Yes / No) between groups were analyzed by Chi-Square test. Correlations and multicollinearity between predictors were analysed, where predictors with correlations higher than 0.80 [6] were excluded from further analysis. Linear regression was used to identify predictors. Analyses were run for each stratified sample individually, while adjusting for other predictors. Since this study only included a limited number of participants that were further divided into ACT and TAU, the number of predictors had to be quite low and carefully selected according to theoretical hypotheses as to why specific variables were important. The present study should be considered exploratory, thus no correction for multiple testing was made. This was decided upon since no similar studies have previously been published, exploring predictors for outcome following ACT specifically targeting body image, in patients with an ED at a specialized ED center.

## Results

### Sample characteristics

In total, 99 participants were included in the RCT; 52 where randomized to ACT, and 47 to TAU. Of those allocated to ACT, five never began treatment and five attended less than half of the sessions before they dropped out. The reasons for dropout differed; two participants became severely ill and needed more intensive treatment, one participant moved, and two dropped out for unknown reasons. In the present study, only participants who completed the intervention were included in the analyses of ACT. Further, one participant was removed from the analyses of TAU since she participated in ACT despite randomization (for clinical and ethical reasons). Further, only participants with assessments at both baseline and follow up at T4 were included. The sample in the present study thus included 37 women in ACT and 40 in TAU. Characteristics of the sample at baseline can be found in Table 1. There were no significant differences between groups regarding baseline characteristics. A t test comparing baseline symptom severity levels for treatment completers to those who dropped out showed that there were no significant differences in EDE-Q at baseline *t*(45) = 0.48, *p* = 0.63. There was no significant difference in baseline severity for those who never began treatment *t*(45) = − 1.51, *p* = 0.14, in comparison to treatment completers. There was a significant difference in severity of MADRS-S at baseline for those who dropped out from treatment, compared to treatment completers, *t*(13.41) = 4.02, *p* < 0.01. This was due to lower ratings of depression in participants who dropped out. There was no significant difference in baseline severity of depression for those who never began treatment *t*(71) = − 0.54, *p* = 0.59, in comparison to treatment completers.

**Table 1:** Characteristics of the sample at baseline

	ACT (*n* = 37)	TAU (*n* = 40)
AN spectrum, *n* (%)	17 (45.9)	22 (55.0)
BN spectrum, *n* (%)	20 (54.1)	18 (45.0)
Age at inclusion, *M(SD)* *Range (min*–*max)*	27.65 (8.16) 18–47	26.45 (7.39) 16–45
Age at onset, *M(SD)* *Range (min*–*max)*	17.00 (5.76) 10–38	15.67 (3.16) 11–29
BMI, *M(SD)* *Range (min*–*max)*	24.10 (6.72) 16.33–48.55	22.69 (5.56) 17.63–41.40
EDE-Q, *M(SD)* *Range (min*–*max)*	3.24 (1.16) 0.84–5.60	3.23 (1.18) 0.18–5.51
Objective binge-eating *M(SD)* *Range (min*–*max)*	4.75 (6.30) 0–20	3.97 (6.45) 0–28
Self-induced vomiting *M(SD)* *Range (min*–*max)*	2.49 (5.47) 0–23	1.79 (4.36) 0–20
Laxative use, *M(SD)* *Range (min*–*max)*	0.97 (4.74) 0–28	0.18 (0.88) 0–5
Compulsive/driven exercise, *M(SD)* *Range (min*–*max)*	5.22 (7.34), 0–28	5.54 (10.81) 0–56
Length of current treatment episode *Range (min*–*max)*	11.89 (10.70) 0–48	13.48 (13.06) 0–63
MADRS-S *Range (min*–*max)*	17.06 (7.31) 4–36	16.56 (8.18) 3–34

ACT, Acceptance and commitment therapy; TAU, Treatment as usual; AN, anorexia nervosa; BN, bulimia nervosa; BMI, body mass index; EDE-Q, eating disorder examination questionnaire

Of the 37 participants who completed the ACT treatment and had data at T4, 11 (29.7%) rated ED symptoms by EDE-Q below cut-off at baseline. At two-year follow-up, 29 (78.4%) of the 37 participants rated ED symptoms by EDE-Q below cut-off, while 23 (62%) rated themselves subjectively as completely recovered. No ACT participant reported deterioration according to RCI (1.45) on the EDE-Q global score. Of the 40 TAU participants, 14 (35%) rated ED symptoms by EDE-Q below cut-off at baseline. At two-year follow-up, 24 (60%) rated ED symptoms by EDE-Q below cut-off, while 15 (37.5%) rated themselves subjectively as completely recovered. Three (7.5%) participants reported deterioration on the EDE-Q global score according to RCI (1.45).

### Outcome, t test

**Fig. 1:**
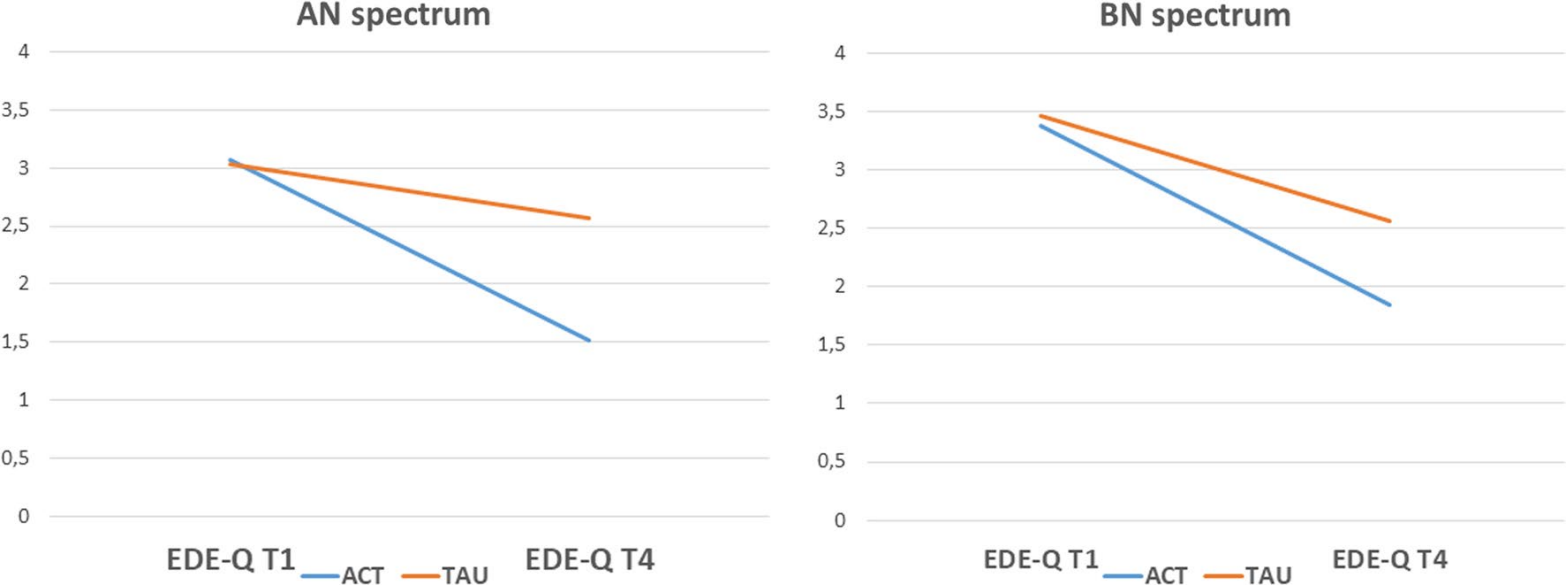
Linear presentation of mean levels of EDE-Q at T1 and T4, for participants with AN or BN spectrum disorders, respectively

Participants in ACT showed a significantly greater change score in EDE-Q from baseline to follow-up compared to participants in TAU (see Table 2). The ACT treatment participants with AN spectrum showed a significantly greater change score reduction in ED symptoms compared to those in TAU (see Fig. 1). There was a significant difference in change score among younger participants, while those who received ACT showed improvement, while participants in TAU showed little change at all. For individuals with a shorter treatment duration before randomization, change score was significantly higher for participants in ACT compared to TAU. There was no significant difference in outcome between groups for participants with different baseline levels of depression. For participants with ratings of EDE-Q below cut-off (2.76) at baseline the change score was significantly higher in the ACT group compared to TAU where participants showed no improvement. There was a significant association regarding subjective experience of recovery and group as participants in ACT were more likely to rate themselves as recovered.

**Table 2:** Change scores on EDE-Q from T1 to T4 in subgroups of participants and distributions on experience of recovery with Chi-Square test

	ACT *M*(*SD*)	TAU *M*(*SD*)	*Change* *t*(df)
Change EDE-Q global score	1.54 (1.34), N = 37	0.66 (1.55), N = 40	− 2.67 (75)**
AN spectrum	1.56 (1.34), N = 17	0.47 (1.55), N = 22	− 2.32 (37)*
BN spectrum	1.53 (1.37), N = 20	0.90 (1.55), N = 18	− 1.33 (36)
Age < 25 at inclusion	1.72 (1.22), N = 19	0.00 (1.39), N = 18	− 4.00 (35)***
Age ≥ 25 at inclusion	1.36 (1.46), N = 18	1.20 (1.48), N = 22	− 0.33 (38)
Treatment duration < 12 months	1.85 (1.32), N = 24	0.73 (1.79), N = 24	− 2.48 (46)*
Treatment duration ≥ 12 months	0.98 (1.23), N = 13	0.57 (1.13), N = 16	− 0.94 (27)
MADRS-S < 20	1.43 (1.41), N = 22	0.62 (1.47), N = 22	− 1.86 (42)
MADRS-S ≥ 20	1.76 (1.34), N = 13	1.10 (1.60), N = 12	− 1.11 (23)
EDE-Q T1 < 2.76	0.90 (1.04), N = 11	− 0.25 (1.24), N = 14	− 2.53 (23)*
EDE-Q T1 ≥ 2.76	1.82 (1.37), N = 26	1.16 (1.49), N = 26	− 1.67 (50)

	N (expected)	N (expected)	*χ*^2^ (df)
Subjectively recovered, YES	23 (18.5)	15 (19.5)	4.27 (1)*
Subjectively recovered, NO	14 (18.5)	24 (19.5)	

ACT, Acceptance and commitment therapy; TAU, Treatment as usual; EDE-Q, eating disorder examination questionnaire

^*^p < .05

^**^p < .01

^***^p < .001

### Outcome, regression

Ratings of EDE-Q and MADRS-S were correlated (*r* = 0.53, *p* < 0.001), however they were below the suggested cut-off of 0.80. Further correlations between the predictors age at inclusion, treatment duration, scores on MADRS-S and EDE-Q ranged from − 0.213 (*p* = 0.063) to 0.199 (*p* = 0.101). Results from the linear regressions, with EDE-Q at T4 as the outcome variable and regressed by intervention type, over stratified samples while controlling for other baseline characteristics, can be found in Table 3. The results showed that diagnosis (AN/BN spectrum) did not predict outcome, while controlling for other variables. Participants that were younger, had a shorter duration of current treatment episode and lower ratings of depression benefitted more from ACT than TAU on symptoms of ED.

**Table 3:** Results from linear regression analysis with EDE-Q at two-year follow-up as outcome variable, for each baseline characteristic between ACT and TAU, while controlling for other characteristics

EDE-Q at T4 between ACT and TAU stratified by	*B* (95% CI)	SE	*p*
AN spectrum	− 0.856 (− 1.721 to .010)	0.423	.052
BN spectrum	− 0.515 (− 1.526 to .496)	0.494	.306
Age < 25 at inclusion	− 1.364 (− 2.268 to − .461)	0.440	.005
Age ≥ 25 at inclusion	− 0.379 (− 1.371 to .612)	0.486	.441
Treatment duration < 12 months	− 0.959 (− 1.833 to − .085)	0.431	.032
Treatment duration ≥ 12 months	− 0.624 (− 1.628 to .381)	0.483	.211
MADRS-S < 20	− 0.884 (− 1.705 to − .063)	0.406	.036
MADRS-S ≥ 20	− 0.560 (− 1.904 to .784)	0.642	.394
EDE-Q T1 < 2.76	− 0.803 (− 1.804 to .197)	0.472	.108
EDE-Q T1 ≥ 2.76	− 0.612 (− 1.507 to .282)	0.443	.174

EDE-Q, eating disorder examination questionnaire; ACT, Acceptance and commitment therapy (set to 1); TAU, Treatment as usual (set to 0); Negative values in beta indicate lower ratings of EDE-QT4 for individuals in ACT

## Discussion

The results from the present study suggests that younger patients, those with shorter treatment lengths, and those with lower ratings of depression are more likely to improve in ED symptoms if they attend ACT compared to TAU. While ACT has shown to be superior to TAU [12], these results add to prior results by showing that some patient characteristics are indicative of greater likelihood of a good outcome from this particular intervention. While this study did not evaluate rapid response, which is often put forward as important for recovery for patients with an ED [29], duration of current treatment was important for outcome. For patients with a longer treatment duration, change scores were lower in both ACT and TAU, indicative of less favorable outcome. This could indicate that for patients who have not achieved an early change, and is not remitted within one year, neither ACT nor TAU is sufficient for achieving a positive outcome.

For participants with an age below 25 at inclusion, the ACT intervention was helpful in reducing ED symptoms, but not for those randomized to TAU who showed no further improvement. For participants at or above 25 years, there was no difference in outcome by treatment condition (ACT or TAU) as both groups improved. The results indicate that ACT-specific processes are especially helpful for young adults. Young adults might be especially susceptible for receiving support from a group of individuals with similar body image issues, which might aid in normalizing body dissatisfaction.

Comorbidity with depression is common in individuals with an ED [21], and is associated with poorer treatment outcome [26]. However, in the present study individuals with more severe symptoms of depression showed improvement on ED symptoms irrespective of treatment type, indicating that both ACT and TAU show potential of improving ED symptoms for individuals with comorbid depression. This could be due to higher motivation for change among participants with more severe ratings. Participants with lower self-rated depressive symptoms at baseline showed only small improvements in ED symptoms if continuing TAU, while reductions were greater for those who received ACT. Accordingly, this could be seen as motivation being lower for patients with lower ratings of depression. However, a body image specific intervention could provide motivation for behavioral change in patients with an ED. These results would be interesting to investigate further, for instance by follow-up measures of depressive symptoms. While the ACT intervention in the present study focused on body image, ACT has been more rigorously investigated for patients with depression, where a review of the empirical evidence for ACT as treatment for depression and anxiety concluded that the effect of ACT was equivalent in outcome to cognitive behavior therapy [40]. Thus, it would be interesting to investigate whether the ACT intervention targeting body image could influence depressive symptoms in addition to the reductions seen in ED symptoms.

ED symptom severity ratings did not show different outcomes between ACT or TAU, while controlling for other characteristics. In another study on ACT for patients with an ED [22] results showed that patients with more severe symptoms at baseline were more likely to benefit from ACT than TAU. In the present study, results did not support this finding since participants with higher scores showed improvement irrespective of treatment type. Results from the present study indicate the benefit of targeted interventions—even for participants who have already quite low ED symptom ratings. The TAU participants with low ratings at baseline showed no further improvement on self-rated ED symptoms. Low self-rated ED symptoms over time could indicate that patients reached a “normative discontent” level of symptoms, and that from a clinical perspective, this might suggest that treatment should be discontinued. However, as stated in the introduction, elevated ratings of body image issues among patients with ED at end of treatment might be interpreted as not having reached full recovery [4], which has also been shown to impair quality of life [27]. By addressing issues with body image in patients with residual ED symptoms, the ACT group continued to improve and reported subjective experiences of remission at a higher rate compared to the TAU group. This is in accordance with a study by Marco et al. [31] which investigated the effect on outcomes when they added a targeted body image intervention to TAU. They reported that the number of sessions was not important for ED symptom improvement, but rather the content of the intervention. They highlighted the importance of including targeted body image interventions to enhance outcome from TAU.

Participants with an AN-spectrum disorder had three times more beneficial change score of ED symptoms when they received ACT compared to TAU, according to the t-tests. Although the regression analysis failed to find statistical significance, the p-value of 0.052 could indicate that the study was underpowered. These results could be worth of continued investigation, given the often-reported treatment-resistant nature in AN patients [1, 15]. Patients with AN often have low motivation for change, which has been described from the perspective that patients see the AN as beneficial. It has been suggested that motivation could be enhanced by including focus on values [15], and body image issues [1, 24]. While other studies have investigated outcome from targeted body image interventions in samples with mixed diagnoses (e.g.: [19, 31]), they did not include analyses of differences between ED diagnoses. Dingemans et al. [8] investigated predictors of different outcome variables for patients receiving ED treatment at a specialized ED center. They concluded that outcome did not differ between patients with AN, BN, BED or EDNOS (DMS-IV). The authors discuss this from the perspective that many patients migrate between diagnoses [33], and irrespective of their specific disorder they share the overvaluation of shape and weight according to a transdiagnostic view [11].

### Strengths and limitations

The method of stratifying the sample into different subgroups poses some limitations that should be considered. The total sample size is small, and further divided by treatment type, thus by stratifying the already small sample into subgroups, each subgroup consists of very few participants. Despite this, we did find some significant differences in the analyses stratified by treatment conditions. The subgroup categories should be easy for clinicians to use as they refer to screening tools commonly used in clinical practice, and treatment duration for comparison of their own patients. Another limitation is that no measure of rapid response was included. However, the current study included participants with varying lengths of current treatment episodes, and prior treatment might have obscured such results. One of the reason for the limited study sample is due to loss of data at follow up, where 11 out of 88 (12.5%) participant failed to fill in measures of outcome. We do not know why these participants refrained from providing data, and how this could affect outcome. A final limitation is the differences between the ACT and TAU conditions. The ACT condition was based on ACT, and targeted body image, while TAU had neither as main content. Thus, there are many aspects that could have led to the different outcomes between groups, which the present study cannot discern.

## Conclusions

The results from the current study indicate that although patients at a group level benefitted more from an ACT group intervention targeting body image versus TAU, younger patients with shorter current treatment length and lower baseline rating of depression benefitted especially from ACT. Furthermore, participants that attended ACT reported a subjective experience of complete recovery at higher rates than participants in TAU did. The study suggests a need for further investigation of patient characteristics that might predict response to body image treatment, particularly regarding ED subtypes and depression ratings.

## Data Availability

The datasets used and/or analysed during the current study are available from the corresponding author on reasonable request.
